# Early High Efficacy Treatment in Multiple Sclerosis Is the Best Predictor of Future Disease Activity Over 1 and 2 Years in a Norwegian Population-Based Registry

**DOI:** 10.3389/fneur.2021.693017

**Published:** 2021-06-17

**Authors:** Cecilia Smith Simonsen, Heidi Øyen Flemmen, Line Broch, Cathrine Brunborg, Pål Berg-Hansen, Stine Marit Moen, Elisabeth Gulowsen Celius

**Affiliations:** ^1^Department of Neurology, Vestre Viken Hospital Trust, Drammen, Norway; ^2^Department of Neurology, Oslo University Hospital, Oslo, Norway; ^3^Institute of Clinical Medicine, University of Oslo, Oslo, Norway; ^4^Department of Neurology, Telemark Hospital Trust, Skien, Norway; ^5^Institute of Health and Society, University of Oslo, Oslo, Norway; ^6^Oslo Centre for Biostatistics and Epidemiology, Research Support Services, Oslo University Hospital, Oslo, Norway; ^7^Multiple Sclerosis Centre Hakadal, Grønvoll, Norway

**Keywords:** multiple sclerosis, disease modifying therapies, no evidence of disease activity, disease activity, treatment decision

## Abstract

**Background:** Moderate and high efficacy disease modifying therapies (DMTs) have a profound effect on disease activity. The current treatment guidelines only recommend high efficacy DMTs for patients with highly active MS. The objective was to examine the impact of initial treatment choice in achieving no evidence of disease activity (NEDA) at year 1 and 2.

**Methods:** Using a real-world population-based registry with limited selection bias from the southeast of Norway, we determined how many patients achieved NEDA on moderate and high efficacy DMTs.

**Results:** 68.0% of patients who started a high efficacy DMT as the first drug achieved NEDA at year 1 and 52.4% at year 2 as compared to 36.0 and 19.4% of patients who started a moderate efficacy DMT as a first drug. The odds ratio (OR) of achieving NEDA on high efficacy drugs compared to moderate efficacy drugs as a first drug at year 1 was 3.9 (95% CI 2.4–6.1, *p* < 0.001). The OR for high efficacy DMT as the second drug was 2.5 (95% CI 1.7–3.9, *p* < 0.001), and was not significant for the third drug. Patients with a medium or high risk of disease activity were significantly more likely to achieve NEDA on a high efficacy therapy as a first drug compared to moderate efficacy therapy as a first drug.

**Conclusions:** Achieving NEDA at year 1 and 2 is significantly more likely in patients on high-efficacy disease modifying therapies than on moderate efficacy therapies, and the first choice of treatment is the most important. The immunomodulatory treatment guidelines should be updated to ensure early, high efficacy therapy for the majority of patients diagnosed with MS.

## Introduction

Multiple sclerosis is a chronic neuroinflammatory disease with onset in mostly young people, and it is the commonest cause of serious physical disability in adults of working age (1). The condition may have a profound impact on quality of life and employment (2). Interferon as a treatment for multiple sclerosis (MS) was first approved in 1996 (3). In 2006, natalizumab was approved as the first high efficacy disease modifying therapy (DMTs) (4), and in the following years more DMTs followed suit. The therapies are divided into moderate efficacy DMTs, with a well-defined safety profile, and high efficacy DMTs, which are more effective but carries higher risk of serious side effects (5). The current European and American treatment guidelines only advise the use of high efficacy drugs for highly active disease (6, 7). Time to EDSS 6 over the past two decades has increased (8). Although DMTs are not the only reason for this development (9), they likely play an important role (10). There are few head to head randomized clinical trials (RCT), so the importance of real-world evidence has been elevated (11).

The concept of “No Evidence of Disease Activity” (NEDA) has been identified as an ambitious tool for measuring efficacy of DMTs (12). NEDA at 1 year is achieved if there is no history of a clinical relapse, no new activity on magnetic resonance imaging (MRI) and no sign of clinical disease progression measured by expanded disability status scale (EDSS) in the past year (13). Although NEDA is by no means a perfect tool (14), limited disease activity in the first few years of diagnosis is widely regarded as a good prognostic sign (15).

The aim of this study was to determine how many patients in a Norwegian population-based real-world study achieved NEDA at 1 and 2 years and examine the impact of initial treatment choice in achieving NEDA.

## Materials and Methods

### Data Collection and Study Population

The BOT-MS (Buskerud, Oslo and Telemark) is a database comprising the complete population of MS patients in the two counties Buskerud and Telemark, and the majority of the patients in the Norwegian capital Oslo (*n* = 3,951). The data were recorded prospectively until 31.12.2017, but retrieved retrospectively by three neurologists specialized in MS between January and December 2018. Detailed information on the database and data collection has previously been published (9).

For this cohort study, we included all patients who had been treated with moderate efficacy DMTs (interferons, glatiramer acetate, teriflunomidem, and dimethyl-fumarate) and/or high efficacy DMTs (natalizumab, fingolimod, or alemtuzumab) for at least 12 months. All patients had access to all disease modifying drugs as all were available and reimbursed since market access in Europe (Table 1). The definition of moderate and high efficacy DMTs was chosen because at the time of market access, fingolimod was considered high efficacy and dimethyl-fumarate was considered moderate efficacy treatment (16). Consequently, that is how they were utilized in the follow-up period. We only included patients started on the treatment in 2006 or after, as 2006 was the first year our population had access to the first high efficacy drug, natalizumab. Only patients with yearly (±2 months) EDSS and MRI were included. Patients with missing or incomplete information and patients with incomplete information precluding determination of NEDA were excluded. For NEDA-status at year 1 we did not include those that discontinued due to side effects or wish for pregnancy, but we included the patients who discontinued the drug before the full 12 months due to lack of efficacy. For NEDA-status in year 2, we included any patient who had been on the drug for at least 24 months, including treatment interruption due to lack of efficacy, but not interruption due to side-effects and wish for pregnancy. The population was divided into three subgroups dependent on previous treatments: first drug, second drug or third drug. When looking at drugs previously used, we included all drugs the patient had taken for at least 3 months. Alemtuzumab was considered effective from the first treatment.

**Table 1:** Disease modifying therapies and year of European Medical Agency (EMA) approval (http://www.emsp.org/about-ms/ms-treatments/#).

**Disease modifying therapy**		**Year of EMA authorization**
Moderate efficacy	Interferons	1995
	Glatiramer acetate	2000
	Teriflunomide	2013
	Dimethyl-fumarate	2014
High efficacy	Natalizumab	2006
	Fingolimod	2011
	Alemtuzumab	2013

We considered any new or enlarging lesions or new gadolinium enhancing lesions on follow-up brain MRI to represent MRI change. For EDSS, we considered any increase in EDSS on at least two consecutive occasions to represent a worsening of EDSS. Only EDSS documented 3 months or more after a relapse were included. Relapses documented in the patients' hospital records were counted as a relapse, regardless of steroid treatment. If we had a negative finding in one of the three components of NEDA, we considered the patient as NEDA fail even though we did not have one or two of the other components (EDSS, MRI and/or relapse).

We created a predictive variable for the future risk of disease activity based on our previous findings in this population (9) and known prognostic risk factors. Age (17–19), sex (17, 18, 20, 21), symptoms at onset (17, 22, 23), involvement of more than one Kurtzke functional system at onset (24), number of relapses within the first 2 years of onset (17, 21, 24–26), findings on a first MRI (20) and EDSS (27) were used, see Table 2. We graded the risk of disease activity based on seven categories of characteristics at the time of diagnosis that are believed to have an impact on future disease activity. Symptom at onset was defined by Kurtzke's functional system (28) and multiple symptoms at onset was defined as symptoms from two or more functional system. We divided the population into low risk for disease activity (0–3 points), medium risk (4–7), and high risk (8–14p). Based on seven categories, the population was divided into three risk groups: low risk for disease activity (1–3 points), medium risk (4–7), and high risk (8–14p). We did not include spinal cord lesions or gadolinium enhancing lesions as this is not done routinely in the clinical practice.

**Table 2:** The risk of disease activity at the time of diagnosis was calculated according to these seven factors.

	**2 points**	**1 point**	**0 points**
Age at diagnosis	>35	18–35	
Gender		Male	Female
Symptom at onset	Motor, brainstem, cerebellar		ON, sensory, other
Multiple symptoms at onset	Yes		No
Multiple relapses before diagnosis	Yes		No
MRI findings at diagnosis	>10 lesions	5–10	<5
EDSS at diagnosis	3.0 or more	2 and 2.5	1.5 or less

*The population was then divided into low risk (1–3 points), medium risk (4–7 points), and high risk (8–14 points). ON, optic neuritis; MRI, magnetic resonance imaging; EDSS, expanded disability status scale*.

The first generation drugs are referred to as injectables (interferon, glatiramer acetate) and were used as a reference category in calculations of odds ratio. Age was dichotomous in the age category with “old” (≥40 years at time of drug initiation) and “young” (<40 years). To investigate the impact of possible changes in prescription practice, and to correct for missing patients, we split the groups into those patients initiated before 2013, and those initiated after 2013. The year 2013 was chosen as this was when teriflunomide, the first oral moderate efficacy drug, became available.

### Statistics

We used IBM SPSS Statistics 25.0 (IBM Corp., Armonk, NY, USA) for data analysis. Differences in continuous variables between two groups were assessed by independent sample *t*-test. Between groups, differences in continuous variables were tested with Student *t*-test for normally distributed data and Mann-Whitney *U*-test for skewed data. The chi-square-test for contingency tables was used to detect associations between categorical variables. Binary logistic regression analysis was used to investigate the association between the treatments and NEDA, and to adjust for possible confounding effects of sex, age at start of medication, time from onset to start of medication and risk group (low, medium and high risk). The results from the regression analysis are presented as odds ratio, adjusted and unadjusted, with 95% confidence intervals (CI). All *p*-values were two-sided and a 5% significance level was used.

### Ethics

This study was approved by the Regional Ethics Committee in Norway (REK 2015/670). One of the conditions for approval was that strict privacy concerns were respected, and that data was not made publicly available. Specific requests regarding data sharing should be directed to the corresponding author.

## Results

**Figure 1:**
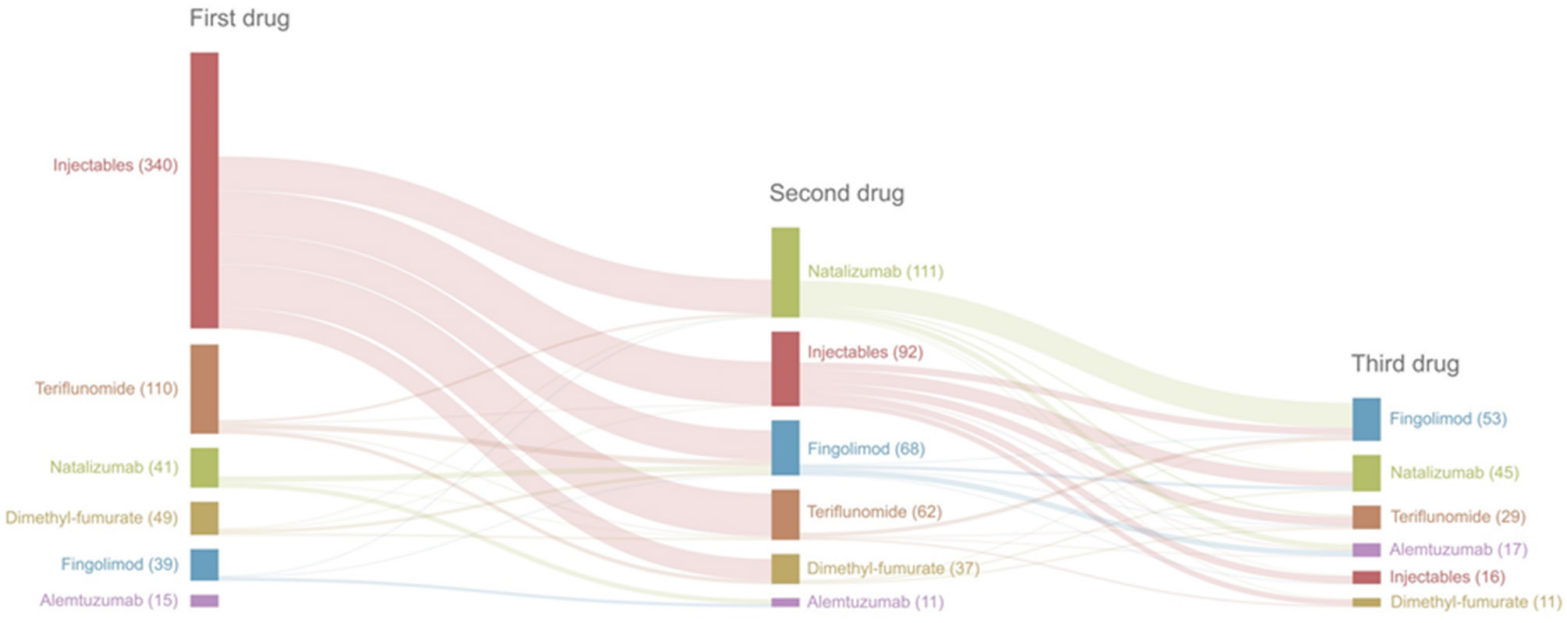
Flow chart of drug swaps.

We included 694 patients with a total of 1,146 drug initiations; demographics are shown in Table 3 and drug swaps are illustrated in Figure 1. Of the patients who started a high efficacy DMT as the first drug, 68.0% achieved NEDA at year 1 and 52.4% achieved NEDA at year 2. Conversely, 36.0% of patients who started a moderate efficacy DMT as a first drug achieved NEDA in year 1 and 19.4% in year 2 (Table 4). The superior effect of high efficacy vs. moderate efficacy DMT on NEDA was highly significant (*p* < 0.001) at both year 1 and 2.

**Table 3:** Demographics of study population.

	**First drug**			**Second drug**			**Third drug**		
	**All**	**Moderate efficacy**	**High efficacy**	**All**	**Moderate efficacy**	**High efficacy**	**All**	**Moderate efficacy**	**High efficacy**
Number of drug initiations	594	491	103	381	191	190	171	56	115
Women, %	67.5	68.4	63.1	69.8^*^	74.3	65.3	70.8	64.3	73.9
Older than 40 years at start, %	47.6	48.9	41.7	54.6	57.1	52.1	59.6^*^	48.2	65.2
Mean age at initiation, years (SD)	38.6 (10.4)	38.8 (10.1)	37.8 (11.9)	40.0 (9.9)	40.8 (9.5)	39.2 (10.2)	40.8 (10.0)	39.8 (11.1)	41.3 (9.4)
EDSS at start, median (IQR)	2.0 (1.0–2.5)	2.0 (1.0–2.5)	1.5 (1.0–2.4)	2.0 (1.5–3.0)	2.0 (1.5–2.5)	2.0 (1.4–2.0)	2.0 (1.5–3.5)	1.5 (1.0–2.0)	2.0 (1.5–2.3)
Mean age at onset, years (SD)	33.8 (9.9)	33.8 (9.6)	33.5 (11.3)	31.8 (9.2)	32.6 (9.0)	30.9 (9.2)	30.3 (9.0)	29.9 (8.7)	30.6 (9.2)
Years from onset to diagnosis, mean (SD)	3.3 (5.3)	3.3 (5.3)	3.2 (4.9)	3.3 (4.9)	3.5 (4.9)	3.2 (4.9)	3.3 (4.1)	2.7 (3.7)	3.5 (4.2)
Years from onset to drug initiation, mean (SD)	4.9 (6.5)	5.0 (6.6)	4.4 (6.1)	8.2 (6.9)	8.2 (6.6)	8.2 (7.2)	10.7 (7.6)	9.8 (8.4)	11.2 (7.1)
Years from diagnosis to drug initiation, mean (SD)	1.6 (3.9)	1.7 (4.0)	1.1 (3.1)	4.9 (4.9)	4.7 (4.5)	5.1 (5.3)	7.3 (5.4)	6.7 (6.0)	7.5 (5.1)
>10 MRI lesions at diagnosis, %	43.6^**^	40.3	59.2	40.7^*^	39.8	41.6	40.4	30.4	45.2
Multiple symptoms at onset, %	30.3	27.7	42.7	30.1	39.8	29.4	32.6	39.1	29.6
≥2 relapses before diagnosis, %	71.9^*^	70.6	78.4	77.3	75.1	79.4	72.8	66.0	75.9
Sensory symptoms at onset, %	40.7^*^	41.1	38.6	40.3	35.3	45.2	43.1	37.0	46.0
Motor symptoms at onset, %	11.8	11.8	11.9	11.2	12.3	10.1	14.4	13.0	15.0
EDSS at diagnosis, median (IQR)	2.0 (1.0–2.0)	2.0 (1.5–2.5)	2.0 (1.0–2.5)	2.0 (1.3–2.0)	2.0 (1.3–3.5)	2.0 (1.0–3.0)	2.0 (1.1–2.0)	1.5 (1.0–2.3)	2.0 (1.5–3.3)
Low risk (0–3 points), %	12.5^**^	14.3	3.9	10.8	12.6	8.9	14.6	21.4	11.3
Medium risk (4–7 points), %	52.2	54.0	43.7	54.9	53.4	56.3	52.6	48.2	54.8
High risk (8–14 points), %	35.4	31.8	52.4	34.3	56.3	34.7	32.7	30.4	33.9

*SD, standard deviation; IQR, interquartile range; EDSS, expanded disability status scale*.

* *p < 0.05*,

** *p < 0.001*.

**Table 4:** NEDA year 1 and NEDA year 2.

		**Year 1**				**Year 2**				
		**Achieved NEDA**			**Total**	**Achieved NEDA**			**Total**	**Missing year 2**
		***n=***	***%***	***p***		***n=***	***%***	***p***		
First drug	Moderate efficacy	177	36.0	<0.001	491	83	19.4	<0.001	428	7
	High efficacy	70	68.0		103	43	52.4		82	3
Second drug	Moderate efficacy	86	45.0	<0.001	191	38	23.5	<0.001	162	5
	High efficacy	127	66.8		190	85	52.1		163	5
Third drug	Moderate efficacy	24	42.9	0.18	56	13	27.1	0.25	48	1
	High efficacy	62	53.9		115	36	36.7		98	2

*Moderate efficacy: Injectables (interferon and glatiramer acetate), teriflunomide and dimethyl-fumarate. High efficacy: Fingolimod, natalizumab, and alemtuzumab*.

The odds ratio of achieving NEDA on a high efficacy DMT as first drug at year 1 was 3.9 (95% CI 2.4–6.1, *p* < 0.001) and at year 2 was 4.6 (96% CI 2.8–7.6, *p* < 0.001) compared to moderate efficacy DMTs (Table 5). The odds ratio did not change meaningfully after adjusting for sex, age at start of medication and time from onset to start of medication. The difference in the proportion of patients achieving NEDA on high efficacy drugs and the odds ratio of achieving NEDA were lower for the second drug, but still highly significant (*p* < 0.001). There was no significant difference for the third drug (Tables 4, 5).

**Table 5:** Odds ratio (OR) analyzed by binary logistics for NEDA year 1 and 2 in high efficacy DMT vs. moderate efficacy DMT, stratified by risk and adjusted for age at initiation of medication, time from onset to initiation of drug and sex.

	**Year 1**				**Year 2**			
	**OR**	**95% CI for OR**		***p-*value**	**OR**	**95% CI for OR**		***p-*value**
		**Lower**	**Upper**			**Lower**	**Upper**	
**First drug**	3.9	2.4	6.1	<0.001	4.6	2.8	7.6	<0.001
Low risk (*n* = 74)	2.3	0.3	18.4	0.44	2.1	0.1	30.5	0.59
Medium risk (*n* = 310)	2.4	1.3	4.6	0.008	3.0	1.4	6.5	0.005
High risk (*n* = 210)	6.2	3.0	13.0	<0.001	5.9	2.8	12.5	<0.001
**Second drug**	2.5	1.7	3.9	<0.001	3.5	2.1	5.6	<0.001
Low risk (*n* = 41)	8.8	1.4	56.6	0.02	12.1	1.6	94.4	0.02
Medium risk (*n* = 209)	2.8	1.6	4.9	<0.001	3.9	2.0	7.7	<0.001
High risk (*n* = 131)	1.9	0.9	4.0	0.07	2.0	0.9	4.5	0.08
**Third drug**	1.5	0.8	2.9	0.25	1.5	0.7	3.4	0.30
Low risk (*n* = 25)	3.3	0.4	28.7	0.28	3.4^*^	0.3	39.3	0.33
Medium risk (*n* = 90)	0.9	0.3	2.1	0.7	1.1	0.4	3.3	0.36
High risk (*n* = 56)	2.2	0.7	7.7	0.2	1.5	0.4	6.3	0.57

*There is minimal difference between unadjusted and adjusted OR*.

* *Not adjusted for gender due to small numbers*.

We also looked at moderate and high efficacy drug initiations before and after 2013 (data not shown), and the findings remained largely unchanged. One exception is that the odds ratio adjusted for initiation before and after 2013 for the second drug increased from 2.5 (95% CI 1.66–3.9, *p* < 0.001) to 3.1 (95% CI 2.0–4.9, *p* < 0.001).

Age did not have a notable impact on the proportion achieving NEDA on the first drug. The proportion of older patients achieving NEDA on a moderate efficacy drug as the second drug was lower than younger patients (37.8 vs. 50.5%), but this was not significant (*p* = 0.08). As a third drug, however, there was a significant difference between moderate and high efficacy drugs in the younger population (72.6 vs. 62.5%, *p* = 0.004), but there was no significant difference in the older age group (Supplementary Tables 1, 2).

Table 6 shows the demographic observations of those who achieved NEDA vs. those who did not achieve NEDA on moderate and high efficacy drugs in the three subgroups. Patients who achieved NEDA on moderate efficacy DMTs were in general slightly older, had longer time from onset to diagnosis and from onset to initiation of treatment. In contrast, this finding tended to be reversed in patients on high efficacy therapies who achieved NEDA. Patients with a medium or high risk of disease activity (87.6% of patients on a first drug, 89.2% of patients on a second drug and 85.3% of patients on a third drug) were significantly more likely to achieve NEDA on a high efficacy therapy as a first drug. There was no significant difference in patients on moderate efficacy therapy, or any second or third drug, regardless of potency.

**Table 6:** Demographics by NEDA and no NEDA.

	**First drug**						**Second drug**						**Third drug**					
	**Moderate efficacy**			**High efficacy**			**Moderate efficacy**			**High efficacy**			**Moderate efficacy**			**High efficacy**		
	**NEDA**	**No NEDA**	***p***	**NEDA**	**No NEDA**	***p***	**NEDA**	**No NEDA**	***p***	**NEDA**	**No NEDA**	***p***	**NEDA**	**No NEDA**	***p***	**NEDA**	**No NEDA**	***p***
	***n* = 177**	***n* = 314**		***n* = 70**	***n* = 33**		***n* = 86**	***n* = 105**		***n* = 127**	***n* = 63**		***n* = 24**	***n* = 32**		***n* = 62**	***n* = 53**	
Women, %	63.8	71	0.10	62.9	63.6	0.94	79.1	70.5	0.18	3.8	68.3	0.54	66.7	62.5	0.75	67.7	81.1	0.10
Older than 40 years at initiation of drug, %	52	47.1	0.30	42.9	39.4	0.74	64.0	51.4	0.08	50.4	55.6	0.50	**66.7**	**34.4**	**0.02**	59.7	71.7	0.18
Age at start, years mean (SD)	**40.2 (9.6)**	**38.0 (10.3)**	**0.02**	38.1 (12.7)	37.3 (9.9)	0.77	41.9 (8.5)	39.8 (10.1)	0.13	39.2 (10.7)	39.2 (9.4)	1.00	**45.2 (9.1)**	**35.7 (10.8)**	**0.001**	40.6 (9.7)	42.1 (9.1)	0.42
EDSS at initiation, median (IQR)	1.8 (1.0, 2.5)	2.0 (1.0, 2.5)	0.87	2.5 (1.5, 3.4)	2.0 (1.5, 2.8)	0.43	2.0 (1.0, 2.5)	2.0 (1.0, 3.0)	0.28	2.5 (2.0, 3.5)	2.5 (1.6, 4.0)	0.64	**2.0 (1.5, 2.5)**	**1.5 (0.8, 2.0)**	**0.01**	2.0 (1.5, 3.5)	2.8 (2.0, 3.5)	0.10
Age at onset, years mean (SD)	34.4 (9.6)	33.5 (9.6)	0.35	33.6 (12.2)	33.2 (9.4)	0.86	32.6 (8.5)	32.7 (9.5)	0.93	31.7 (9.3)	29.4 (9.0)	0.11	32.6 (9.1)	27.8 (7.9)	0.04	31.4 (9.2)	29.7 (9.2)	0.32
Years from onset to diagnosis, mean (SD)	**4.1 (5.8)**	**2.8 (5.0)**	**0.01**	3.3 (5.4)	3.2 (3.7)	0.93	3.8 (5.5)	3.3 (4.4)	0.44	2.7 (4.8)	4.1 (5.1)	0.07	3.1 (4.1)	2.4 (3.3)	0.51	3.1 (3.8)	4.1 (4.6)	0.21
Years from onset to drug initiation, mean (SD)	**5.8 (7.1)**	**4.5 (6.3)**	**0.03**	4.4 (6.1)	4.2 (6.0)	0.65	**9.3 (7.6)**	**7.3 (5.6)**	**0.05**	**7.3 (6.7)**	**10.0 (7.9)**	**0.02**	**12.5 (9.5)**	**7.8 (6.9)**	**0.03**	10.1 (6.0)	12.4 (8.0)	0.08
Years from diagnosis to drug initiation, mean (SD)	1.7 (3.8)	1.7 (4.1)	0.87	1.2 (2.8)	1.0 (3.6)	0.91	**5.4 (5.2)**	**4.1 (3.8)**	**0.05**	4.8 (5.1)	5.8 (5.7)	0.24	**9.4 (6.9)**	**4.7 (4.4)**	**0.003**	6.8 (4.3)	8.3 (5.9)	0.10
Multiple symptoms at onset, %	27.5	27.9	0.93	47.5	32.1	0.17	35.1	27.4	0.28	29.1	30.2	0.89	47.6	32	0.28	30.9	27.9	0.75
>10 MRI lesions at diagnosis, %	**41.9**	**58.1**	**<0.001**	**75.4**	**24.6**	**<0.001**	**55.3**	**44.7**	**<0.001**	65.8	34.2	0.41	41.2	58.8	0.19	57.7	42.3	0.61
≥2 relapses before diagnosis, %	72.2	68.7	0.57	79.1	76.7	0.79	71.4	78.5	0.28	78.8	80.6	0.77	54.5	75	0.13	74.1	78	0.64
Sensory symptoms at onset, %	36.3	43.7	0.11	37.1	41.9	0.65	33.7	36.5	0.69	45.6	44.4	0.88	37.5	36.7	0.95	**35**	**58.5**	**0.01**
Motor symptoms at onset, %	11.7	11.9	0.95	14.3	6.5	0.26	12.0	12.5	0.93	7.2	15.9	0.06	16.7	10	0.47	20	9.4	0.12
EDSS at diagnosis, median (SD)	1.5 (1.0, 2.0)	2.0 (1.0, 2.5)	0.40	1.8 (1.0, 3.5)	2.0 (0.0, 2.3)	0.35	**2.0 (1.0, 2.0)**	**2.0 (1.5, 2.5)**	**0.04**	2.0 (1.5, 3.0)	2.0 (1.0, 2.5)	0.54	1.5 (1.0, 2.3)	1.0 (1.0, 2.5)	0.52	2.0 (1.5, 2.4)	2.0 (1.0, 2.0)	0.63
Low risk of disease activity, %	27.1	72.9	0.09	50.0	50.0	0.43	50.0	50.0	0.60	**64.7**	**35.3**	**0.05**	25.0	75.0	0.16	46.2	53.8	0.55
Medium risk of disease activity, %	37.0	63.0	0.64	**57.8**	**42.2**	**0.05**	40.0	59.8	0.15	64.5	35.5	0.43	55.6	44.4	0.06	52.4	47.6	0.72
High risk of disease activity, %	38.5	61.5	0.45	**77.8**	**22.2**	**0.03**	50.8	49.2	0.25	65.2	34.8	0.72	35.3	64.7	0.45	59.0	41.0	0.44

*SD, standard deviation; IQR, interquartile range; EDSS, expanded disability status scale. Significant findings are highlighted in bold*.

**Figure 2:**
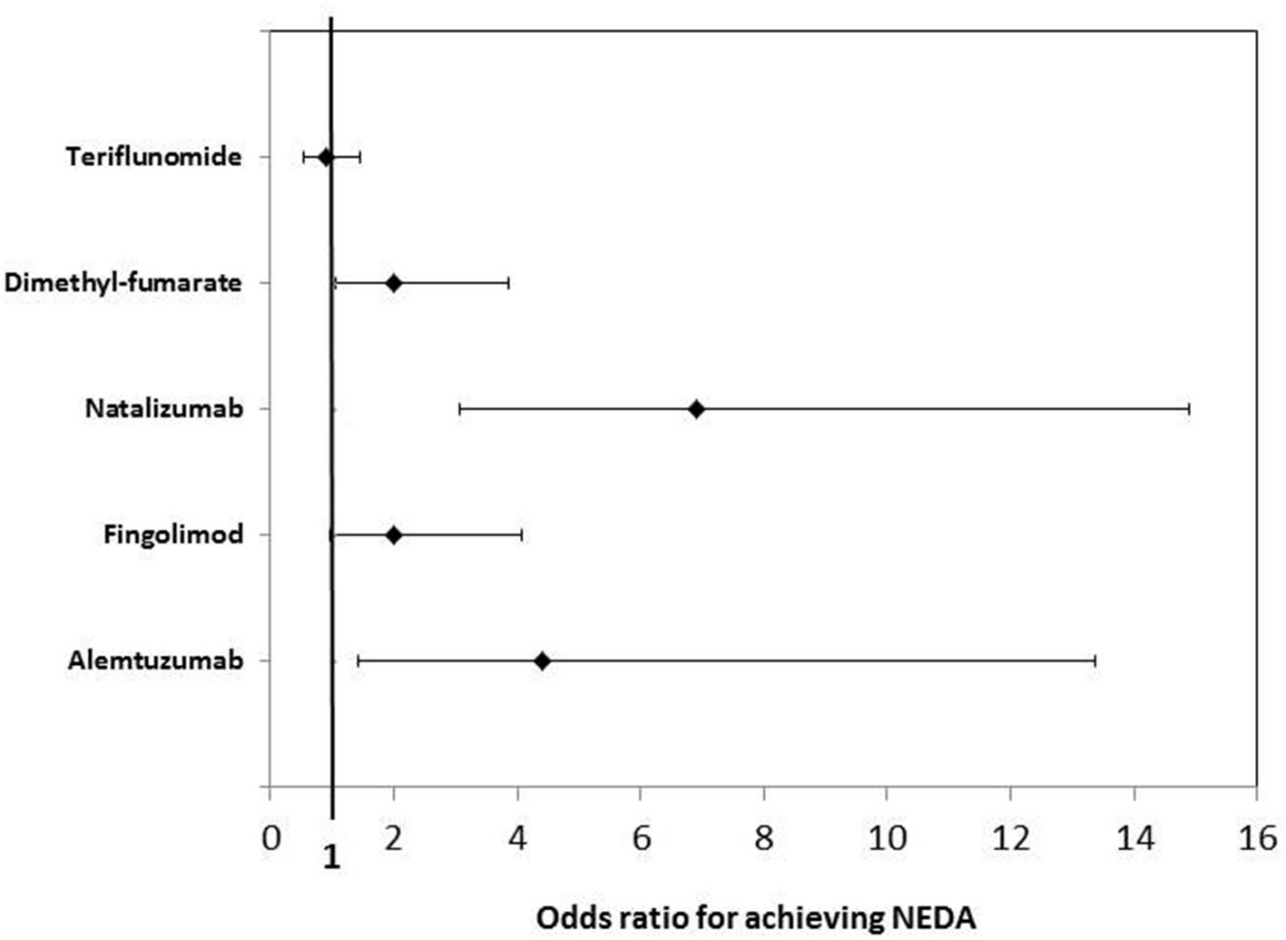
Forest plot of odds ratio for reaching NEDA at year 1 compared to the injectables (interferon and glatiramer acetate).

The numbers of patients on the individual drugs achieving NEDA are presented in Table 7. Natalizumab and fingolimod are the only DMTs that are significantly more likely than the injectables to achieve NEDA at year 1 and 2 as a first drug, though the numbers of alemtuzumab were small and 100% of patients on alemtuzumab as a second drug achieved NEDA. All the DMTs were superior to the injectables as a second drug. The adjusted odds ratio of each individual drug vs. the injectables are presented in Table 7 and Figure 2. Natalizumab as a first drug has an odds ratio of 7.4 (95% CI 3.5–15.4, *p* < 0.001) for reaching NEDA, which is superior to all the other drugs (see Figure 2), though the confidence interval is large. Teriflunomide and dimethyl-fumarate as a first drug did not have significantly better odds ratios at year 1 or 2 than the injectables. As a second drug, all the DMTs were superior to injectables at year 1 and 2. The odds ratio of achieving NEDA on a third drug was less convincing. Adjusting for sex, age at start of medication, time from onset to start of medication and risk groups did not meaningfully alter the results.

**Table 7:** NEDA and odds ratio of reaching NEDA on individual drugs at year 1 and 2.

		**Year 1**						**Year 2**						
		**NEDA**					**Odds ratio**	**NEDA**					**Odds ratio**	**Missing year 2**
		***n*=**	**%**	***p***	**Total**	**OR**	**(95% CI), *p***	***n*=**	**%**	***p***	**Total**	**OR**	**(95% CI), *p***	
First drug	Injectables	118	34.7		340	1.0		61	20.3		301	1.0		3
	Teriflunomide	39	35.5	0.89	110	0.9	(0.6–1.6), *p =* 0.94	11	12.0	0.07	92	0.5	(0.3–1.0), *p =* 0.07	2
	Dimethyl-fumarate	20	48.8	0.08	41	2.0	(1.0–3.8), *p =* 0.05	11	31.4	0.13	35	2.0	(0.9–4.3), *p =* 0.09	2
	Natalizumab	39	79.6	<0.001	49	6.9	(3.2–14.4), *p <* 0.001	27	64.3	<0.001	42	6.5	(3.2–13.1), *p <* 0.001	2
	Fingolimod	21	53.8	0.02	39	2.0	(1.0–4.0), *p =* 0.04	12	40.0	0.01	30	2.4	(1.1–5.3), *p =* 0.03	1
	Alemtuzumab	10	66.7	0.01	15	4.4	(1.5–13.5), *p =* 0.009	4	40.0	0.13	10	2.8	(0.8–10.5), *p =* 0.12	0
	**Total**	**247**	**41.6**		**594**			**126**	**24.7**		**510**			**10**
Second drug	Injectables	30	32.6		92	1.0		11	13.6		81	1.0		0
	Teriflunomide	34	54.8	0.006	62	2.7	(1.4–5.3), *p =* 0.005	13	28.3	0.04	46	2.5	(1.0–6.3), *p =* 0.04	4
	Dimethyl-fumarate	22	59.5	0.005	37	3.3	(1.5–7.6), *p =* 0.004	14	40.0	0.001	35	4.4	(1.7–11.1), *p =* 0.002	1
	Natalizumab	72	64.9	<0.001	111	4.2	(2.3–7.7), *p <* 0.001	54	51.4	<0.001	105	6.5	(3.1–13.7), *p <* 0.001	0
	Fingolimod	44	64.7	<0.001	68	4.4	(2.2–8.7), *p <* 0.001	26	49.1	<0.001	53	6.2	(2.7–14.6), *p <* 0.001	4
	Alemtuzumab	11	100.0	<0.001	11	^*^	^*^	5	100.0	<0.001	5	^*^	^*^	1
	**Total**	**215**	**55.9**		**381**			**123**	**27.8**		**325**			**10**
Third drug	Injectables	4	25.0		16	1.0		1	7.1		14	1.0		0
	Teriflunomide	12	41.4	0.27	29	1.9	(0.5–7.7), *p =* 0.38	7	26.9	0.14	26	4.4	(0.5–40.8), *p =* 0.20	1
	Dimethyl-fumarate	8	72.7	0.01	11	8.5	(1.3–53.7), *p =* 0.02	5	62.5	0.005	8	20.9	(1.7–260.6), *p =* 0.02	0
	Natalizumab	27	60.0	0.02	45	4.4	(1.2–17.1), *p =* 0.03	19	47.9	0.007	40	12.5	(1.4–107.9), *p =* 0.02	1
	Fingolimod	24	45.3	0.15	53	1.9	(0.5–7.0), *p =* 0.36	13	27.7	0.11	47	3.7	(0.4–33.0), *p =* 0.24	1
	Alemtuzumab	11	64.7	0.02	17	4.9	(1.0–24.3), *p =* 0.05	4	36.4	0.07	11	6.9	(0.6–83.6), *p =* 0.13	0
	**Total**	**86**	**50.3**		**171**			**49**	**33.6**		**146**			**3**

*P-values and odds ratio compared to injectables. Odds ratio, analyzed by binary logistics regression, was adjusted for age at start of medication, time from onset to start of drug, sex and risk group*.

* *100% of patients achieved NEDA. The total number and percentage of drugs as a first, second or third drug are highlighted in bold*.

Unsurprisingly, patients on moderate efficacy therapy as a first drug were more likely to discontinue treatment than patients on a high efficacy therapy as a first drug (65.2 vs. 29.2%, *p* < 0.001). This was also the case in patients on a second drug (55.4 vs. 42.7%, *p* = 0.02) but not in patients on a third drug (42.9 vs. 29.5%, *p* = 0.10). Table 8 shows the number of patients who discontinued therapy on moderate and high efficacy therapies and causes of discontinuation. Patients on moderate efficacy therapy as a first drug were more likely to discontinue due to side effects than patients on high efficacy therapy as a first drug (45 vs. 14%, *p* = 0.002). This was also the case for the second drug (40 vs. 14%, *p* < 0.001). The number of patients who discontinued a third drug were too small to draw a conclusion (*n* = 4 and *n* = 5).

**Table 8:** Information on drug discontinuation.

	**First drug**		**Second drug**		**Third drug**	
	**Moderate efficacy**	**High efficacy**	**Moderate efficacy**	**High efficacy**	**Moderate efficacy**	**High efficacy**
Discontinued, *n*=	276 (65.2%)	28 (29.2%)	93 (55.4%)	79 (42.7%)	21 (42.9%)	33 (29.5%)
Months on drug before disontinuation (SD)	32.1 (23.9)	33.7 (21.1)	29.0 (17.0)	48.3 (27.4)	28.4 (17.0)	41.5 (24.6)
**Causes for discontinuation, %**						
Lack of efficacy	31.0	17.9	36.6	10.1	52.4	18.2
Side effects or fear of side effects	44.9	14.3	39.8	13.9	19.0	15.2
Pregnancy	7.2	7.1	5.4	12.7	9.5	6.1
Lack of compliance	5.4	3.6	9.7	6.3	4.8	9.1
Converted to progressive disease	2.9	10.7	4.3	12.7	4.8	12.1
NAB positive	7.6	0	3.2	1.3	4.8	3.0
JCV positive	0	46.4	0	43.0	0	33.3
Unknown	0.4	0	1.1	0	4.8	3.0

*SD, standard deviation; NAB, neutralizing antibodies; JCV, John Cunningham virus*.

## Discussion

In this Norwegian population-based, real-world study we found that patients who start high efficacy therapies are significantly more likely to achieve NEDA at year 1 and 2 than patients starting moderate efficacy therapy. However, the odds ratio of achieving NEDA is reduced for each attempted drug.

Patients started on a high efficacy drug as a first DMT had an odds ratio of achieving NEDA of 3.9 compared to the moderate efficacy drugs, adjusting for sex, age and time from onset to diagnosis. The odds ratio was reduced to 2.5 as a second drug, and the odds ratio of 1.5 was not significant for the third drug. Age did not have a notable impact on the proportion of patients achieving NEDA on the first and second drug, but older patients were less likely to achieve NEDA on the third drug. Our findings illustrate the importance of choosing the most effective drug at the time of diagnosis. These findings were especially strong in the 90% of patients who were classified as having a medium to high risk of disease activity.

NEDA is by no means a perfect tool as it is overly reliant on MRI (29), it does not take into account subtle deterioration in fine motor skills and cognitive changes, and there is no consensus regarding the definitions of the different components (13). Failure to achieve NEDA is not necessarily a good predictor of long-term disability (14). However, neuronal injury occurs early in the disease, and limited disease activity within the first few years of diagnosis is widely regarded as a good prognostic sign (15).

Our findings are in accordance with studies supporting high efficacy therapy at the time of diagnosis compared to an escalation approach (30, 31). The escalation approach may be inadequate to prevent unfavorable outcomes in a real-world population (32), and this is important as the disease activity in the first couple of years influence the disease course (33, 34). The risk of progression at 10 years is highly dependent on EDSS score at 5 years, and it progresses more rapidly from EDSS 4 onwards compared to EDSS 2 and onwards (35). In the absence of a cure, an increasing body of evidence supports early initiation of high efficacy disease modifying treatment in MS to halt disease activity and reduce disability progression (36, 37).

However, many neurologists still utilize a stepwise approach in initiating disease modifying therapy, starting with the safer, but less effective therapies, and only escalate once there is sign of disease activity (38). This is reflected in national guidelines, regulatory bodies and insurance policies (1, 30, 39). In addition, some argue that there is no need for high efficacy treatment in patients with positive prognostic factors and a suspected “mild” disease (5). In our cohort, patients who achieved NEDA on moderate efficacy drugs tended to be older and have longer time from onset and diagnosis to start of drug initiation. This most likely reflects the disease rather than the drug efficacy. Patients with delayed drug initiation after onset and diagnosis have more likely been followed with a watchful wait approach (40). These patients have fewer relapses and less MRI activity, and thus less disease activity and less incentive to initiate immunomodulatory treatment early. However, the concept of mild or benign MS is controversial (37, 41). One study found only nine of 1,049 patients with disease duration of >15 years and EDSS <4 were truly benign (42). Ellenberger et al. found one in four patients with benign MS at 15 years were unemployed, and only one in three remained benign after 30 years (43). Smestad et al. found that although only one third of MS patients in an Oslo cohort had mild disability based on EDSS, half of them were cognitively impaired (44).

One argument for not initiating high efficacy treatment early is the safety profile (15). However, natalizumab has few side effects beyond the risk of progressive multifocal leukoencephalopathy (PML), and this risk has been mitigated with intensified follow-up regimes, monitoring of the JC-virus index and possibly extended interval dosing (45). The three hospitals included in this study utilize natalizumab frequently. We check JC-virus index biannually and discontinue the drug in cases of elevated titres. Due to risk stratification, none of the hospitals has experienced PML, despite a combined population of more than 2,500 patients, or a quarter of the national MS population. In addition, patients treated with alemtuzumab are monitored closely for 5 years after treatment initiation, and there have been no deadly outcomes from alemtuzumab treatment. Also moderate efficacy drugs are certainly not without side effects that can significantly affect quality of life (46). Our population was significantly more likely to discontinue moderate efficacy therapies due to side effects than high efficacy therapies. The injectables have poorer acceptability profiles than other DMTs, and the high efficacy drugs have lower dropout rates than moderate efficacy drugs (47). Although side effects from moderate efficacy therapies are rarely life threatening, there are several reported cases of PML in Tecfidera treated patients (48). In the end, higher disability at a younger age seems a more significant risk than most of the adverse effects associated with established high efficacy DMTs.

The European (ECTRIMS/EAN) guidelines of 2018 suggest the choice of treatment depends on patient characteristics, disease severity, safety profile and drug accessibility (6). They advise escalating treatment if there is disease activity despite injection therapy. The American Academy of Neurology guidelines notes that patients with a highly active disease should be treated with high efficacy DMT (7). Neither the European guidelines, nor the American guidelines recommend a specific treatment strategy. Two large randomized clinical trials (TREAT-MS, NCT035300328 and DELIVER-MS, NCT03535298) examining escalation vs. early high efficacy therapy are currently underway and will provide valuable information on the short-term differences between these two treatment strategies. However, the differences in long-term disability will require decades of follow-up time, and the available evidence favors early high efficacy therapy. In our opinion, international guidelines should consider updating their recommendations according to current knowledge.

The strength of this study is the well-defined study population. The ratio of neurologists per capita in 2017 was 9.5/100,000 (data from The Norwegian Doctors' Union), and almost all Norwegian MS patients are followed by neurologists at public hospitals. There are few neurologists in private practice. All Norwegian MS neurologists had complete access to all therapies available in Europe at the time of approval, and all these drugs are reimbursed. Real-world studies, such as this, are not restricted by stringent inclusion criteria but instead assess the entire heterogeneous population and can therefore be generalized beyond their study frames (49). BOT-MS is a population-based registry, and a major strength as a real-world study is that we have limited selection bias and know who is missing and why.

Real-world data is also subject to missing data, which is a source of potential information bias. Many of the patients started on the injectables might not have been followed as strictly as those started on the newer drugs. Thus, patients with enough information on the composites of NEDA to be included were likely to have more disease activity. This means there may be an underrepresentation of NEDA patients in this group. This possible information bias was partly counteracted by only including patients started on treatment as of 2006, the year the first highly potent disease modifying drug, natalizumab, was made available to our patients. From this point onwards, there was a more stringent follow-up process of all MS patients. In addition, the odds ratio for teriflunomide was the same as injectables. Our findings also remained largely unchanged before and after 2013, which marks the introduction of teriflunomide and dimethyl-fumarate.

We have created a risk score to categorize patients as having low, medium and high risk of disease activity. Our choices in creating this score were based on available literature (17–26), though we acknowledge that others may categorize the risk differently. In fact, the MS community's ability of predicting individual disease development is limited (37). Our score is based on easily accessible data, though ideally it should have included information on smoking (50), vitamin D (51), and spinal cord lesions, gadolinium enhancing lesions (52) and atrophy (53) on MRI, to name a few. This score has not yet been validated, and we would like to validate it in a new population. We could have used propensity score analyses to control for confounding, but propensity score matching does not yield different estimates compared to conventional multivariate methods (54) and is often used inappropriately in MS research (55).

We acknowledge that treatment allocation bias may play a role in this study. The cohort exposed to high efficacy drugs as a first drug were younger, with lower disease duration and more MRI lesions and relapses at presentation. It is likely that this would lead to a greater response to immunotherapy (15). We do not believe this weakens our study, but rather strengthens our findings and our conclusion that more people should be offered high efficacy therapies. Of the 199 people with medium or high risk of disease activity diagnosed in 2013 or after, 64% were started on moderate efficacy therapy as a first drug. These patients should have received high efficacy therapy from the start (37).

We have chosen to categorize fingolimod as a high efficacy therapy since that is how it was portrayed when it first arrived on the market (56). International, national and local guidelines (6) consequently recommended it as a choice for treatment escalation in highly active MS during the span of this study, and treatment choices were subsequently decided based on this premise. However, many studies conclude that fingolimod has a similar efficacy profile to the moderate efficacy therapy dimethyl-fumarate (57, 58), though not all (16, 47). We have shown NEDA-data on each individual drug in this study in addition to the two efficacy groups. Regardless, the allocation of fingolimod as a moderate efficacy therapy would only strengthen our conclusion that achieving NEDA is significantly more likely in patients on high-efficacy disease modifying therapies.

Another potential bias is observation bias. All patients treated with natalizumab are seen monthly, and the patients treated with teriflunomide are seen frequently in the first year after initiation. These patients were thus more inclined to mention relapses to their treating MS team, as opposed to the remaining MS patients who are seen less often (59). Despite this, natalizumab patients did better than other patients. Another weakness is the retrospective data retrieval, subjecting the study to investigator bias. This was ameliorated by only having three neurologists specialized in MS to include in the database based on a mutually accepted manual. Finally, we did not have enough observations to make a confident statement on the odds ratio of reaching NEDA on alemtuzumab, and we have not included cladribine, another high efficacy DMT, which was approved after the inclusion period.

## Conclusion

Achieving NEDA is significantly more likely in patients on high-efficacy disease modifying therapies than on moderate efficacy therapies, and the first choice of treatment is the most important. Moderate efficacy therapies should be used with caution in most MS patients, unless the clinician is confident the patient has a less active form of MS. There is a need for updating immunomodulatory treatment guidelines ensuring early, high efficacy therapy for the majority of patients diagnosed with MS.

## Data Availability Statement

The datasets presented in this article are not readily available because one of the conditions for ethics approval was that strict privacy concerns were respected, and that data was not made publicly available. Requests to access the datasets should be directed to cecsim@vestreviken.no.

## Ethics Statement

The studies involving human participants were reviewed and approved by Regional Ethics Committee in Norway (REK 2015/670). Written informed consent for participation was not required for this study in accordance with the national legislation and the institutional requirements.

## Author Contributions

CS, HF, LB, PB-H, SM, and EC: design and conceptualization. CS, HF, and LB: data acquisition. CS, HF, LB, CB, PB-H, SM, and EC: data analysis, data interpretation, and writing original draft. All authors contributed to the article and approved the submitted version.

## Conflict of Interest

CS has received unrestricted research grant from Sanofi and Novartis, advisory board and/or speaker honoraria from Sanofi, Merck, Novartis, and Biogen Idec. HF has received unrestricted research grant Biogen Idec and Novartis, advisory board and/or speaker honoraria Sanofi, Merck, and Biogen Idec. LB has received an unrestricted grant from Sanofi Genzyme and advisory board honoraria from Merck. PB-H has received advisory board and/or speaker honoraria from Novartis, UCB, Sanofi, Merck, and Biogen Idec. EC has received unrestricted research grants from Novartis and Sanofi, advisory boards and/or speaker honoraria (Almirall, Biogen Idec, Merck, Roche, Novartis, Sanofi, and Teva). The remaining authors declare that the research was conducted in the absence of any commercial or financial relationships that could be construed as a potential conflict of interest.
