# Supplementary material for: Early High Efficacy Treatment in Multiple Sclerosis Is the Best Predictor of Future Disease Activity Over 1 and 2 Years in a Norwegian Population-Based Registry
Source: Front Neurol. 2021 Jun 17;12:693017. doi: 10.3389/fneur.2021.693017 (PMC8248666; doi:10.3389/fneur.2021.693017)
Supplement: Supplementary file 1 [file Data_Sheet_1.PDF]

Supplementary tables

			Year 1							Year 2						
			NEDA			Total	OR		NEDA			Total	OR	
			n=	%	*p*		(95% CI)	*p*	n=	%	*p*		(95% CI)	*p*
**First drug**	Young	Moderate efficacy	85	33.9	*<0.001*	251	*0.25*		42	19.3	*<0.001*	218	*0.22*	
		High efficacy	40	66.7		60	(0.14-0.46)	*<0.001*	24	51.5		47	*(0.11-0.43)*	*<0.001*
	Old	Moderate efficacy	92	38.3	*<0.001*	240	*0.27*		41	19.5	*<0.001*	210	*0.21*	
		High efficacy	30	69.8		43	(0.13-0.54)	*<0.001*	19	54.3		35	*(0.10-0.44)*	*<0.001*
**Second drug**	Young	Moderate efficacy	31	37.8	*<0.001*	82	*0.27*		10	15.2	*<0.001*	66	*0.12*	
		High efficacy	63	69.2		91	(0.14-0.52)	*<0.001*	47	60.3		78	*(0.05-0.27)*	*<0.001*
	Old	Moderate efficacy	55	50.5	*0.04*	109	*0.55*		28	29.2	*0.03*	96	*0.53*	
		High efficacy	64	64.6		99	(0.31-0.97)	*0.04*	38	44.7		85	*(0.28-0.99)*	*0.05*
**Third drug**	Young	Moderate efficacy	8	72.4	*0.004*	29	*0.25*		6	22.2	*0.13*	27	*0.50*	
		High efficacy	25	62.5		40	(0.08-0.77)	*0.02*	13	40.6		32	*(0.15-1.70)*	*0.27*
	Old	Moderate efficacy	16	59.3	*0.38*	27	*1.47*		7	33.3	*0.90*	21	*0.79*	
		High efficacy	37	49.3		75	(0.59-3.64)	*0.41*	23	34.8		66	*(0.27-2.35)*	*0.67*

Supplementary table 1: *NEDA1 and NEDA2 by age (young: <40 years old at start of drug. old: ≥40 at start of drug), adjusted for years from onset to start of drug and sex. OR odds ratio, CI confidence interval*

			Year 1							Year 2						
			NEDA			Total	OR			NEDA			Total	OR	
			n=	%	*p*		(95% CI)	*p*		n=	%	*p*		(95% CI)	*p*
First drug	Moderate efficacy	Young	85	33.9	*0.30*	251	0.9			42	19.3	0.95	218	1.0	
		Old	92	38.3		240	(0.6-1.4)	*0.74*		41	19.5		210	(0.6-1.7)	*0.92*
	High efficacy	Young	40	66.7	*0.74*	60	0.9			24	51.1	0.77	47	1.0	
		Old	30	69.8		43	(0.4-2.1)	*0.78*		19	54.3		35	(0.4-2.5)	*1.0*
Second drug	Moderate efficacy	Young	31	37.8	*0.08*	82	0.7			10	15.2	0.04	66	0.5	
		Old	55	50.5		109	(0.3-1.3)	*0.24*		28	29.2		96	(0.2-1.2)	*0.12*
	High efficacy	Young	63	69.2	*0.50*	91	1.0			47	60.3	0.05	78	1.7	
		Old	64	65.6		99	(0.5-1.9)	*0.94*		38	44.7		85	(0.8-3.3)	*0.14*
Third drug	Moderate efficacy	Young	21	72.4	*0.02*	29	0.4			6	22.2	0.39	27	0.8	
		Old	16	59.3		27	(0.1-1.4)	*0.14*		7	33.3		21	(0.2-4.0)	*0.81*
	High efficacy	Young	25	62.5	*0.18*	40	1.2			13	40.6	0.58	32	0.9	
		Old	37	49.3		75	(0.5-2.9)	*0.66*		23	34.8		66	(0.3-2.3)	*0.82*

***Supplementary table 2**: NEDA year 1 by age (young: <40 years old at start of drug, old: ≥40 at start of drug), adjusted for years from onset to start of drug and sex. Missing year 2= not enough information on MRI, relapses or EDSS to ascertain NEDA status in year. OR odds ratio, CI confidence interval*
